# Administration Routes as Modulators of the Intrahepatic Distribution and Anti-Anemic Activity of Salicylic Acid/Fe3O4 Nanoparticles

**DOI:** 10.3390/biomedicines10051213

**Published:** 2022-05-23

**Authors:** Bogdan Mîndrilă, Sandra-Alice Buteică, Ion Mîndrilă, Dan-Eduard Mihaiescu, Marina-Daniela Mănescu, Ion Rogoveanu

**Affiliations:** 1 Doctoral School, University of Medicine and Pharmacy of Craiova, 200349 Craiova, Romania; bogdanmindrila01@gmail.com (B.M.); manescu_marina@yahoo.com (M.-D.M.); 2 Faculty of Pharmacy, University of Medicine and Pharmacy of Craiova, 200349 Craiova, Romania; alice.buteica@umfcv.ro; 3 Department of Morphology, Faculty of Medicine, University of Medicine and Pharmacy of Craiova, 200349 Craiova, Romania; 4 Faculty of Applied Chemistry and Materials Science, University Politehnica of Bucharest, 011061 Bucharest, Romania; danedmih@gmail.com; 5 Department of Gastroenterology, Faculty of Medicine, University of Medicine and Pharmacy of Craiova, 200349 Craiova, Romania; ionirogoveanu@gmail.com

**Keywords:** iron oxide nanoparticles, salicylic acid, administration routes, bleeding-induced anemia, liver zonation

## Abstract

The liver is a key organ in the pharmacokinetics of iron oxide nanoparticles (IONPs). This paper examined how the intravenous (IV) or intragastric (IG) route of administration influenced the intrahepatic distribution or therapeutic effects of IONPs. Wistar rats, some with bleeding-induced anemia, and iron oxide nanoparticles functionalized with salicylic acid (SaIONPs), with an average hydrodynamic diameter of 73 nm, compatible with rat sinusoid fenestrations, were used in this study. Light microscopy and multispectral camera analysis of Prussian blue labeled SaIONPs allowed mapping of intrahepatic nanoparticle deposits and revealed intrahepatic distribution patterns specific to each route of administration: loading of Kupffer cells and periportal hepatocytes when the IV route was used and predominant loading of hepatocytes when the IG route was used. Reducing the time to return to baseline values for hemoglobin (HGB) in rats with bleeding-induced anemia with IV or IG therapy has proven the therapeutic potential of SaIONPs in such anemias. The long-term follow-up showed that IV therapy resulted in higher HGB values. Proper use of the administration routes may modulate intrahepatic distribution and therapeutic effects of nanoparticles. These results may be beneficial in theragnosis of liver disease.

## 1. Introduction

Iron oxide nanoparticles (IONPs) can be easily designed and possess favorable physicochemical properties that make them very attractive platforms for use in diagnostic and therapeutic medical fields, such as imaging [1,2,3], cancer therapy [4,5,6,7,8], regenerative medicine and tissue engineering [9,10,11], cell labeling [12], gene therapy [13], and iron replacement therapy [14,15,16]. Surface coating and functionalization allow the modulation of the IONPs stability, biocompatibility, and toxic activity [11,17,18,19,20]. To date, few commercial IONPs approved for clinical use have been withdrawn over time [21], and this requires further IONPs pharmacokinetics studies [20,22]. Biodistribution and interference with the physiological metabolism of iron are aspects of IONPs pharmacokinetics, which needed to be studied to understand their potential toxicity [23].

Hepatic uptake is the most prominent way to retain IONPs from the bloodstream [20,24], especially when their size is between 10 to 15 nm and 200 nm [15]. Preferential liver accumulation [25] makes IONPs versatile agents in theragnosis of liver diseases [6,26]. Their retention in the liver has been described in Kupffer cells as the main site of phagocytosis [27], endothelial cells [28], and hepatocytes [24,29]. IONPs distribution in the liver compartments can influence the rate of liver metabolism [28] and, therefore, their effects on the hepatic disease.

In addition to size, the route of administration may influence the distribution of IONPs in the liver compartments [24,30]. Regardless of the route of administration (i.e., intravenous, oral, inhalation, intraperitoneal, etc. [24,31]), IONPs reach the liver through the bloodstream. The liver has two distinct blood supplies represented by the portal vein, which provides about 75% of the blood flow, and the hepatic artery is responsible for 25% of the blood flow [32]. Blood from the portal venules and smaller branches of the hepatic artery enters the sinusoids and further reaches the central venules, larger hepatic veins, and vena cava [33]. Liver lymphatic fluid formed by the plasma component filtered through the fenestrae of the sinusoids passes into Disse spaces, delimited by sinusoids and hepatocytes [34], and then into Mall spaces located around the portal tract, to enter the lymphatic vessels [35]. The portal vein drains blood from the gastrointestinal tract [33] and is the main pathway through which the IONPs are transported to the liver after oral or intraperitoneal administration [30]. Intravenous or inhaled IONPs initially enter the pulmonary circulation and then reach the liver through the hepatic artery [30], loading mainly the liver, spleen, and lung [24].

Salicylic acid functionalized IONPs (SaIONPs) have shown good biocompatibility in avian [36,37] or murine [30] models. Although intravenous and intraperitoneal injection are the main administration routes for IONPs [31], SaIONPs have been shown to be suitable for oral [38] and gavage [30] administration.

In this paper, we studied the distribution pattern of SaIONP deposits in the liver compartments in relation to intragastric (IG) or intravenous (IV) routes used for their administration. We also evaluated the route-related therapeutic effects of SaIONPs in rats with bleeding-induced anemia. These results may be beneficial in theragnosis of liver disease.

## 2. Materials and Methods

### 2.1. Functionalized Nanoparticles Aqueous Dispersion

SaIONPs with an average hydrodynamic diameter of 73 nm, a polydispersity index of 0.14, a zetapotential of +50.5 mV, and an iron concentration of 0.49 mg/mL were used in this study. These functionalized nanoparticles have been synthesized through a modified Massart method and characterized by FTIR, DLS, and ICP–MS analyses, as some of us have previously reported [38]. Previously we estimated that 1 mL of SaIONPs contained 0.67 mg Fe3O4 and about 0.46 mg salicylic acid.

### 2.2. Animals

In this study, 42 Wistar white rats from the Biobase of the University of Medicine and Pharmacy of Craiova were used. The rats were kept in plastic cages in suitable ambient conditions (T = 23 ± 1 °C; H = 50 ± 5%; light/dark 12:12 h) and unlimited access to water and standard food. Protocols involving the use of animals were approved by the Ethics Committee of the University of Medicine and Pharmacy of Craiova (139/20 December 2019).

Twelve rats weighing 342.3 ± 18.9 g were randomly divided into 3 groups. The first 2 groups received 1 mL of SaIONPs (3.3 mg/kg) administered either IG (*n* = 5) by gavage or IV (*n* = 5) in the tail vein. Rats in group 3 (*n* = 2) were not treated, constituting the control group. After 24 h, the rats were sacrificed and autopsied to harvest the digestive tract viscera for the microscopic evaluation of the SaIONPs distribution.

Fifteen male rats weighing 506 ± 70 g were used to assess the dry weight and total iron content of feces excreted after IG or IV SaIONPs administration. After laboratory accommodation, feces excreted by rats were harvested twice a day for 2 days. Then the rats were randomly divided into three groups, and each received a dose of 1 mL SaIONPs (2.2 mg/kg) administered IG (IG group, *n* = 5) or IV (IV group, *n* = 5), respectively, a dose of 1 mL of fresh-water-administered IG (control group, *n* = 5). The excreted feces were collected from each group for another 2 days. All the rats were sacrificed and autopsied 7 days after nanoparticles administration to evaluate the SaIONPs intrahepatic distribution. The feces were dried at 70 °C for 24 h and manually grounded into a fine powder with a mortar and pestle.

Fifteen male rats weighing 309 ± 25.9 g were subjected to daily bleeding via phlebotomy for twelve consecutive days until 92.2 ± 1.4% of the estimated total blood volume (ETBV) was harvested. Every blood sampling was performed via ventral tail artery puncture under Sevorane^®^ anesthesia. The first samples were used to determine basal levels of hematological parameters. Daily blood samplings were kept under 10% of ETBV to minimize the risk of hypovolemic shock. The bleeding was followed by an equivalent volume of normal saline solution delivered via intraperitoneal injection. All procedures were performed under aseptic conditions. The total blood volume of a rat was estimated by using the following formula: ETBV = animal’s weight (g) × 0.064 [39]. On day 14, rats with bleeding-induced anemia were randomly divided into three groups: untreated control group (*n* = 5), IG-treated group (*n* = 5), and IV-treated group (*n* = 5). Treated groups received 3 doses of 1 mL SaIONPs (3 × 3.6 mg/kg) administered IG and IV, respectively, on experiment days 14, 15, and 16. An additional 0.3 mL of blood samples was collected at days 14, 21, 28, 35, and 42 for hematological parameters assessment in the post-therapy recovery phase. A BC-5000 Vet Auto Haematology Analyser was used to determine values of the following hematological parameters in the blood samples: red blood cell count (RBC), hemoglobin concentration (HGB), and hematocrit (HCT). All the rats were sacrificed and autopsied on day 42 of the experiment to evaluate the SaIONPs intrahepatic distribution.

### 2.3. Histopathology Analysis

After euthanasia, the liver and viscera of the gastrointestinal tract were harvested separately, fixed for 48 h in 10% neutral buffered formalin, and processed for paraffin embedding. Seriate sections that were 5 µm thick were cut on a Microm HM355 rotary microtome from each tissue block and collected on poly-L-lysine coated slides. For light microscopy, the sections were stained with H&E and Perls Prussian blue. Rabbit anti-GFAP (Z0334, diluted as 1:15.000, Dako, Glostrup, Denmark) and goat anti-iba-1 (ab5076, diluted as 1:800, Cambridge, UK) primary antibodies were used for single enzymatic immunohistochemistry. Briefly, the deparaffinized sections were processed for antigen retrieval (microwaving in citrate buffer pH 6 for 21 min), for blocking the endogenous peroxidase activity (30 min incubation in 1% hydrogen peroxide), immersion in 3% skimmed milk in PBS (30 min), and then incubation with the primary antibodies at 4 °C (18 h). The signal was amplified with a species-specific peroxidase polymer–based system adsorbed for rat immunoglobulins (Nikirei-Bioscience, Tokyo, Japan) for 30 min and then was detected with 3,3′-diaminobenzidine (Dako). A series of slides were counterstained with hematoxylin and coverslipped in DPX (Sigma-Aldrich, St. Louis, MO, USA), while another series was further stained for 30 min at 45 °C in a 1% acidic potassium ferrocyanide solution (Perls Prussian blue) for visualizing ferric iron, counterstained with nuclear red, and coverslipped in DPX.

A quantitative analysis of SaIONP deposits was performed with a Nuance analysis software (Perkin Elmer, Hopkinton, MA, USA) on images taken with a Nuance FX multispectral camera attached to a Nikon Eclipse 90i system (Elta90, Bucharest, Romania). After building a spectral library from individual slides stained with either DAB, Perls Prussian blue, and nuclear red, we were able to efficiently unmix and characterize the SaIONP deposits in the hepatocytes and Kupffer cells. Perls Prussian blue–stained liver sections were used to measure the relative area (RA) of SaIONP deposits in liver acini: RA = SaIONP deposits’ area/measured area. For each evaluated liver, twenty rectangular areas that were 100 pixels wide and had a variable length, delimited by portal spaces and neighboring central spaces, were randomly selected on multispectral camera images. The selected rectangular areas were binarized (Image-Pro Plus 6.0) and divided into 10 bands (b_n_) of equal length (length of b_n_ = length of rectangular area/10). The RA of SaIONP deposits was calculated for each band of rectangular area: RAb_n_ = number of colored pixels in b_n_/(length of b_n_ × 100). Bands b_1_, b_2_, and b_3_ were associated with periportal zone (zone 1); bands b_4_, b_5_, and b_6_ were associated with intermediate zone (zone 2); and bands b_7_, b_8_, b_9_, and b_10_ were associated with pericentral zone (zone 3) of the hepatic acinus.

### 2.4. ICP–OES Analysis

The total iron content of the feces was measured by using Echelle polychromator/CID-TE cooled array detector (Thermo Scientific, Cambridge, UK). Experimental conditions: ICP–OES Thermo, iCAP 6000, iTeva software (version 2.7.0.87, Thermo Scientific, Cambridge, UK), plasma power 1150 W, radial plasma view, peristaltic pump speed 50 rpm, nebulizer flow 1.5 L/min, toroidal nebulizer, plasma argon flow 12 L/min, result in 3 readings, and calibration for 23 elements according to the data table, with 1 analytical spectral line and 2 validation spectral lines. Homogenized fine fecal powder (1.5–2 g) from each sample was placed in a melting pot and dried at 105 °C for 7 h. After cooling and weighing, the material was placed in the calcination oven at 550 °C for 5 h. The resulting ash was treated with 3 mL of nitric acid and diluted with ultrapure water. The result of the ICP–OES analysis was expressed in the dry mass.

### 2.5. Statistical Analysis

The results are presented as the mean and standard deviation (SD) for all the variables. Data were compared by using a one-way ANOVA with Tukey’s post hoc analysis or Student’s *t*-test, and the significance level was set at 0.05. Statistical analysis was performed by using SPSS version 16 (SPSS, Inc., Chicago, IL, USA).

## 3. Results

### 3.1. Mapping the Distribution of SaIONPs in Liver and Gastrointestinal Tract

**Figure 1:**
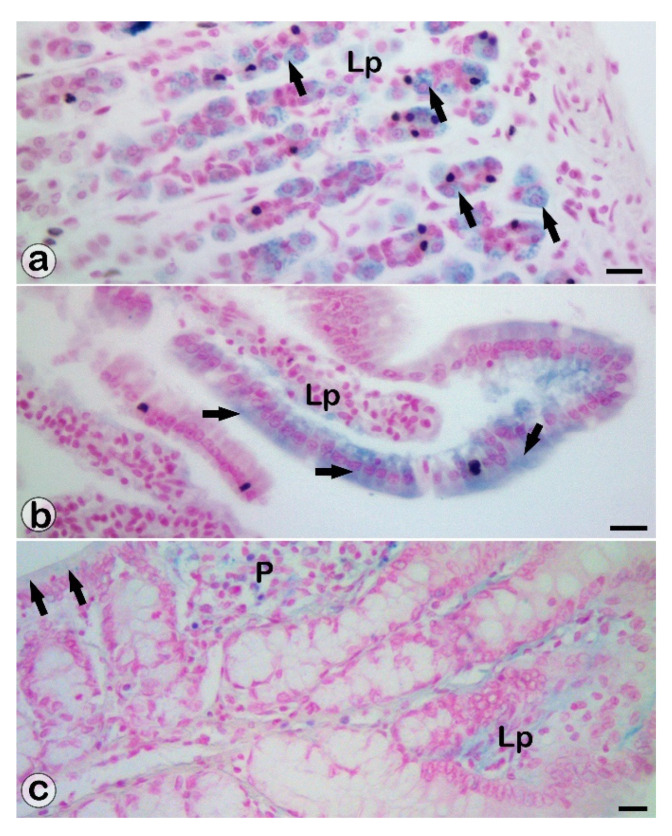
SaIONP deposits (blue colored) in the gastrointestinal-tract walls. Microscopy highlighted SaIONPs in lamina propria (Lp) and epithelium (black arrows) of the basal regions of the gastric glands (**a**), intestinal villi (**b**), Peyer’s patches (P), and glands of the proximal colon (**c**). Viscera harvested 24 h after IG administration of SaIONPs. Perls Prussian blue stainings. Bar = 10 µm.

After IG administration, the SaIONPs were identified as intracytoplasmic deposits in the parietal cells of the basal regions of the gastric glands, in the enterocytes, and in the mucosal cells of the colon. In addition, SaIONP deposits were observed not only in the mucosal lamina propria and capillaries but also in the submucosal vessels of the stomach, small intestine, and proximal colon (Figure 1).

**Figure 2:**
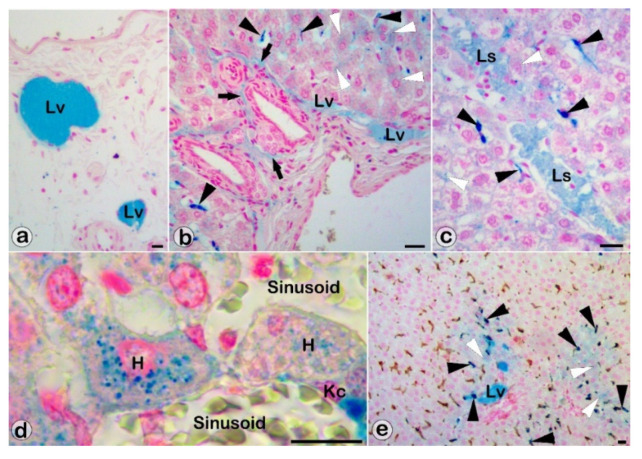
SaIONP deposits in liver compartments: lymphatic vessels (Lv) of the hepatic hilum (**a**), lymphatic vessels and connective tissue (black arrows) of the Moll space (**b**), interlobular sacciform lymphatic spaces (Ls) (**c**), and Kupffer cells (Kc, black arrowheads) and hepatocytes (H, white arrowheads) (**b**–**e**). Viscera harvested 24 h after IG (**a**–**d**) or IV (**e**) SaIONPs administration. Perls Prussian blue (**a**–**d**) and anti-Iba-1 (**e**) stainings. Bar = 10 µm.

The microscopic analysis of the Perls Prussian blue–stained liver sections revealed SaIONP deposits in all compartments of the rat hepatic liver, regardless of the SaIONPs administration routes. Immunohistochemistry associated with Perl’s Prussian blue staining identified SaIONP deposits in Iba-1^+^ cells, but not in GFAP^+^ cells. SaIONPs that have reached the liver can be retained by Kupffer cells and endothelial cells of sinusoidal capillaries, can pass through sinusoids into central veins and further into the systemic venous circulation, or can pass into Disse spaces. SaIONPs from the Disse spaces can be retained by hepatocytes or can pass into Moll spaces and intrahepatic lymph vessels to leave the liver via the lymphatic pathway (Figure 2).

Sacciform lymphatic spaces (Figure 2c) located at the periphery of the hepatic lobules, between the portal tracts, became visible and easily identifiable under light-microscopy due to their dilation caused by SaIONPs’ accumulation in the liver lymphatic compartment.

No SaIONP deposits in the bile ducts were identified on the liver sections of the treated rats. Moreover, no SaIONP deposits were identified on the sections through the liver, stomach, small intestine, or colon harvested from rats in the control group.

**Figure 3:**
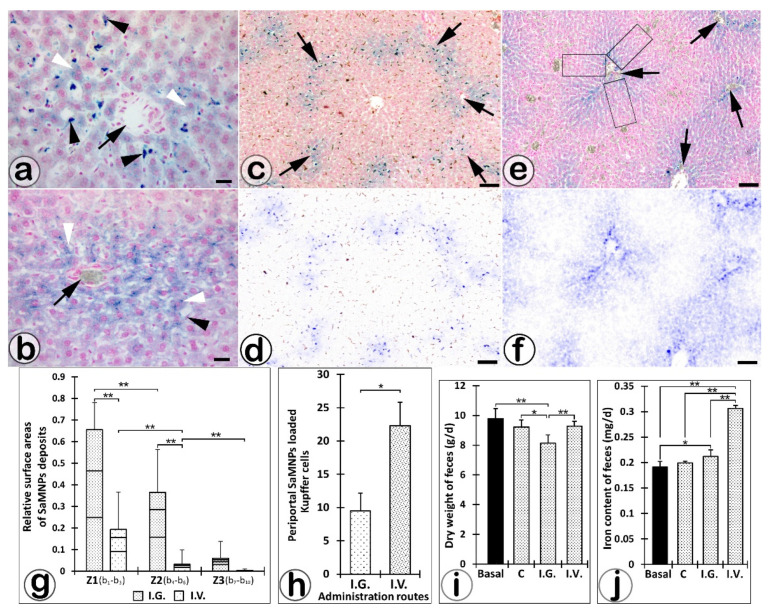
Microscopy of the intracytoplasmic SaIONP deposits in hepatocytes (white arrowheads) and Kupffer cells (black arrowheads) located around the portal areas (black arrows) of the liver parenchyma harvested 24 h after IV (**a**) or IG (**b**) SaIONPs’ administration. The SaIONPs’ specific loading patterns of hepatic acini after IV (**c**,**d**) or IG (**e**,**f**) administration revealed by multispectral camera analysis (the boxes indicate the selected and analyzed areas around a portal space). Different zonal distribution of SaIONP deposits in periportal areas (**g**) and periportal Kupffer cells (**h**) relative to IV or IG routes of SaIONPs administration. Variation of dry weight (**i**) and total iron content of the feces after the IV or IG (**j**) SaIONPs’ administration (* *p* < 0.05; ** *p* < 0.01; (**g**–**i**) one-way ANOVA with post hoc Tukey test; (**h**) Student’s *t*-test). Perls Prussian blue (**a**,**b**,**c**,**e**) and anti-Iba-1 (**c**,**d**) stainings. Bar = 10 µm (**a**,**b**); 50 µm (**c**–**f**).

Microscopy of the Perls Prussian blue stained liver sections showed specific patterns of SaIONPs’ loading of liver acini (Figure 3a–f), as confirmed by multispectral camera analysis, which showed statistically significant differences (one-way ANOVA with post hoc Tukey test) in the distribution of SaIONP deposits in the compartments of liver acini related to IG or IV routes used for nanoparticles’ administration.

The IG route of administration was associated with gradual accumulation of SaIONP deposits in hepatocytes from the periportal zone to the pericentral zone of the hepatic acini (Figure 3a,b), with (RA periportal zone)/(RA intermediate zone) = 1.8 (*p* < 0.01) and (RA intermediate zone)/(RA pericentral zone) = 6.2. IV administration was followed by the accumulation of most SaIONP deposits in periportal Kupffer cells and hepatocytes, with (RA periportal zone)/(RA intermediate zone) = 6 (*p* < 0.01) and (RA intermediate zone)/(RA pericentral zone) = 9.3 (*p* < 0.01). In addition, the number of periportal Kupffer cells with intracytoplasmic SaIONP deposits was about 3-fold higher (Student’s *t*-test, *p* < 0.01) when the IV route was used (Figure 3h).

The RA of SaIONP deposits in livers harvested 24 h after IG or IV treatment was 3.4-fold higher in periportal zone (*p* < 0.05), 11.4 -fold higher in intermediate zone (*p* < 0.05), and 17-fold higher in pericentral zone of hepatic acini when the IG route was used (Figure 3g).

**Figure 4:**
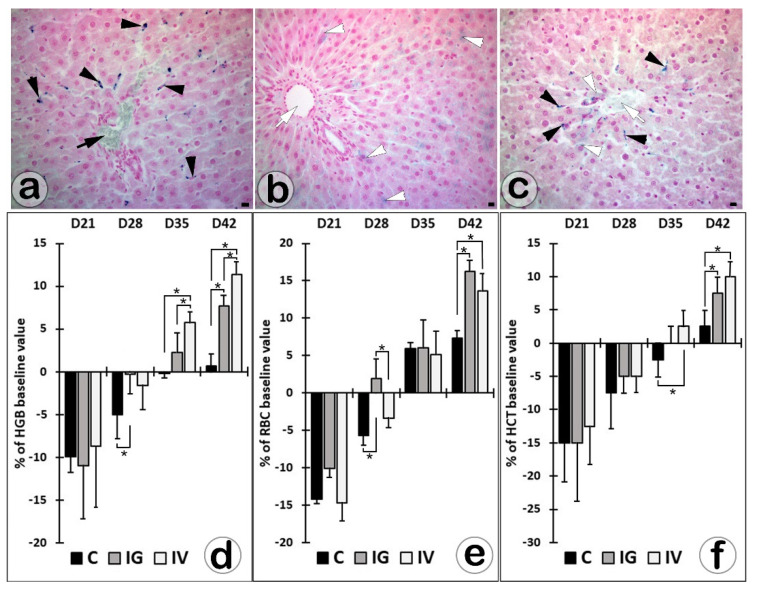
Kupffer cells (black arrowheads) and hepatocytes (white arrowheads) with intracytoplasmic SaIONP deposits in the portal (black arrows) and central (white arrows) spaces 7 days after IV (**a**) or IG (**b**,**c**) nanoparticles’ administration (Perls Prussian blue staining; Bar = 10 µm). Post-therapeutic values and time of return to baseline values for HGB (**d**), RBC (**e**), and HCT (**f**) in untreated (C) or IV/IG-treated rats with bleeding-induced anemia (* *p* < 0.05; one-way ANOVA with post-hoc Tukey test).

A successive set of 20 portal and central spaces were examined by light-microscopy on each section made by livers harvested 7 days after IG or IV SaIONPs’ administration. After IV administration, 36.4 ± 7% of the portal spaces were found to contain nanoparticle deposits, located in the Kupffer cells’ cytoplasm (Figure 4a). Most portal spaces no longer contained SaIONP deposits visible under light microscopy in IG administration (Figure 4b). In this situation, nanoparticle deposits were observed in 6.6 ± 4% of central areas, both in Kupffer cells and in hepatocytes (Figure 4c). No SaIONP deposits were observed in the hepatic lobules after 26 days of treatment.

### 3.2. Feces Analysis

A significant decrease in the dry weight of daily excreted feces was observed in rats treated IG with 1 mL SaIONPs (8.13 g ± 0.54) compared to rats treated IV with the same SaIONPs dose (9.27 g ± 0.31, *p* < 0.01) or those in the control group treated IG with 1 mL saline (9.22 g ± 0.47, *p* < 0.05) (Figure 3i). The ICP–OES analysis showed a significant increase in iron content of feces in IV-treated rats (0.3 mg ± 0.005, *p* < 0.01) compared to IG treated rats (0.21 mg ± 0.013) or control rats (0.2 mg ± 0.003, *p* < 0.01) (Figure 3j).

### 3.3. SaIONPs Effects in Bleeding-Induced Anemia

The baseline values of HGB, RBC, and HCT were 154.5 ± 3.9 g/L, 7.26 ± 0.15 (10^12/L), and 0.40 ± 0.01. Their values after bleeding anemia induction were 115.3 ± 8.6 g/L, 5.21 ± 0.22 (10^12/L), and 0.29 ± 0.02, respectively. On day 42, the mean values of HCT, HGB, and RBC in post-therapeutic and control groups showed statistically significant differences (*p* < 0.05), and all were higher than the corresponding baseline values. Moreover, the HGB level was significantly higher in IV-treated than in IG-treated rats (Figure 4d–f).

The HGB and HCT returned to baseline between days 28 and 35 of the experiment in the IG- and IV-treated rats and between 35 and 42 in the control rats. The RBC count returned to baseline between days 21 and 28 of the experiment in IG-treated rats and between 28 and 35 in the control and IV-treated mice (Figure 4d–f).

## 4. Discussion

Functional and structural compartmentalization of the rat liver still has multiple approaches, and controversy over this topic remains outside the scope of this study [32,33,40,41,42]. In this paper, both the classical concept of the hepatic lobules described by Kiernan for the analysis of the compartments of the hepatic tissue and the concept of the hepatic acinus described by Rappaport for the functional zonation of the hepatic tissue were used. The hepatic lobule has a polygonal shape and consists of liver structures disposed around an efferent central venule. Each corner of this polygon is formed by a portal triad consisting of a portal vein, hepatic artery, and bile duct [43]. The liver acinus is the area of liver tissue supplied individually by the portal vessels [44] with zonal functional heterogeneity [41,42].

Specific features of the hepatic compartments are among the factors that modulate the liver SaIONPs’ loading. Kupffer cells are circulating macrophages that polarize after adherence to the sinusoids [45] and are mainly responsible for phagocytizing IONPs in the liver [24]. It has been observed that Kupffer cells are more numerous and have a higher phagocytic activity in zone one (periportal) than in zone two (midzonal) and zone three (perivenos) of the rat liver acinus [46]. Before forming a complex three-dimensional network [47], the periportal sinusoids have portions perfused only by the branches of the portal vein or only by the branches of the hepatic artery, because these branches open separately into the sinusoids [48]. The common pathway for sinusoidal entry of both SaIONPs carried by the hepatic artery and circulating macrophages may explain the predominant loading of periportal Kupffer cells observed after IV administration of SaIONPs.

Higher perfusion pressure [49] and larger endothelial fenestrations [50,51] of the portal extremities are traits of the rat sinusoids that can be linked to the SaIONPs specific liver distribution after IG administration. Due to these features, the SaIONPs carried by the branches of the portal vein can pass more easily into the Diesse spaces of the periportal zone, thus decreasing probability of SaIONPs uptake by Kupffer cells. The SaIONPs from the Diesse spaces can be taken up by hepatocytes or can increase the lymphatic flow, with consequent dilation of interlobular, subcapsular, or Moll space lymphatic collectors. The inverted pattern distribution observed in 7-day-old IG-treated livers, in which most SaIONP deposits were present in the pericentral hepatocytes and Kupffer cells, showed that nanoparticles filtered in the periportal Disse spaces may return to sinusoids in pericentral area. Kupffer cells are more numerous in the periportal sinusoids than in the central ones [52]. Furthermore, the SaIONPs’ retention profiles highlighted by us in the rat periportal areas suggest that the Kupffer cells were more numerous in the segments of sinusoids supplied only by the branches of the hepatic artery than in segments of sinusoids supplied only by the branches of the portal vein.

The oral route of nanoparticles’ administration is attractive due to the greater convenience and high patient compliance [53]. The main obstacles encountered in oral administration are the acidity of the gastric environment, which can affect the stability and solubility of nanoparticles, and the mucus barrier, which can block the penetration and absorption of nanoparticles [54]. We observed that SaIONPs have easily overcome these obstacles and have proven to be versatile for oral administration.

Identification in the lamina propria and capillaries of the stomach, small intestine, and proximal colon showed that SaIONPs were not affected by the pH variations and were absorbed in all segments of the gastrointestinal tract. The high rate of absorption can explain why total iron in the feces does not increase, as we observed in the group of rats with IG administration of SaIONPs. A post-therapeutic decrease in the dry weight of feces excreted by rats in this group can be attributed to constipation, a gastrointestinal side effect also observed with other oral iron supplements [55].

In the absence of active excretory mechanisms, intestinal absorption of iron is strictly controlled to avoid the toxic effects of iron overload [56]. This study showed that SaIONPs’ retention in Kupffer cells was higher in IV administration. Iron ions resulting from intracytoplasmic SaIONPs’ degradation can induce the upregulation of ferritin [57], which is one of the main actors in the hepatic mechanism of the intestinal iron absorption control [58]. Therefore, the rapid loading and degradation of SaIONPs by Kupffer cells may explain the increase in total fecal iron, as observed in IV-treated rats.

IONPs have proven their potential in the oral and IV management of anemia, and increasing the HGB concentration in the organism was one of their post-therapeutic benefits [15,59,60]. The results of this study showed that the SaIONPs’ administration in rats with bleeding-induced anemia shortened the time to return to baseline values of the hematological parameters investigated (HGB, RBC, and HCT) and ultimately produced their statistically significant increases over the values recorded in the group of untreated rats. Preferential retention in Kupffer cells, which release iron ions from IONPs faster [28], may be associated with the higher increase in HGB values observed in the SaIONPs-treated rats on IV route.

The short-term exposure to IONPs’ administered by gavage at doses of 25 mg/kg (three consecutive days) has not been associated with adverse histological effects or significant enzymatic changes in mouse liver [61]. Minimal cytotoxic effects were also reported in rats that received IONPs on IV route for 4 consecutive weeks at doses of 7.5 mg/kg/week [62]. No adverse effects on the liver were observed with oral administration of 4.8 mg/kg/day salicylic acid for 13 weeks [63]. Because the doses of SaIONPs used in these experiments were less than 3.6 mg/kg/day, they can be considered safe, with no toxic effects on the liver.

We noted that the SaIONPs are homogeneously distributed in the lymphatic and interstitial compartments, without forming large distinct clusters in light microscopy. Therefore, the nanoparticles accumulated in these compartments determined their uniform blue coloration. Nanoparticle cell loading can be performed either passively or by adsorbed or receptor-mediated endocytosis [64]. In passive uptake, internalized SaIONPs are not surrounded by membranes, and Perl’s staining will show a uniform distribution of nanoparticles in the cytoplasm of these cells. Internalization by endocytosis is followed by the formation of intracellular vesicles filled with SaIONPs, which, in the Perl’s stain, will have the appearance of coarse deposits.

## 5. Conclusions

This study showed a dependence between the intrahepatic distribution of the SaIONP deposits and the IV/IG routes of their administration. The IV route of administration favored the uptake of SaIONPs by periportal Kupffer cells and hepatocytes, while the IG route favored their predominant uptake by hepatocytes. This study also highlighted the therapeutic potential of SaIONPs in bleeding-induced anemia. Although both routes of administration have shortened the time to return to baseline values for HGB, longer-term follow-up showed that the IV route of SaIONP administration was followed by higher increases in HGB values. Proper use of routes of administration may modulate intrahepatic distribution and therapeutic effects of nanoparticles. These results may be beneficial in the theragnosis of liver disease.

## Data Availability

All data are present in the main text.
